# A Combination of Podophyllotoxin and Rutin Alleviates Radiation-Induced Pneumonitis and Fibrosis through Modulation of Lung Inflammation in Mice

**DOI:** 10.3389/fimmu.2017.00658

**Published:** 2017-06-09

**Authors:** Savita Verma, Bhargab Kalita, Sania Bajaj, Hridayesh Prakash, Ajay Kumar Singh, Manju Lata Gupta

**Affiliations:** ^1^Institute of Nuclear Medicine and Allied Sciences (INMAS), DRDO, New Delhi, India; ^2^Laboratory of Translational Medicine, School of Life Sciences, University of Hyderabad, Hyderabad, India

**Keywords:** podophyllotoxin, rutin, pneumonitis, pulmonary fibrosis, inflammation, reactive oxygen species

## Abstract

Pneumonitis and pulmonary fibrosis are predominant consequences of radiation exposure, whether planned or accidental. The present study, demonstrates radioprotective potential of a formulation, prepared by combining podophyllotoxin and rutin (G-003M), in mice exposed to 11 Gy thoracic gamma radiation (TGR). Treated mice were observed for survival and other symptomatic features. Formation of reactive oxygen species (ROS)/nitric oxide (NO) was measured in bronchoalveolar lavage cells. DNA damage and cell death were assessed in alveolar cells by terminal deoxynucleotidyl transferase dUTP nick-end labeling assay. Total protein (TP), lactate dehydrogenase (LDH), and alkaline phosphatase (ALP) were measured in bronchoalveolar lavage fluid (BALF)/serum of mice to assess lung vascular permeability. Interleukin-6 (IL-6), tumor necrosis factor-α (TNF-α), transforming growth factor-β1 (TGF-β1), cluster of differentiation 45, inducible nitric oxide synthase (iNOS), and nitrotyrosine were also estimated in lungs/BALF of differentially treated mice. Our observations revealed 100% survival in G-003M-pretreated mice against 66.50% in 11 Gy TGR exposed. Other symptoms like reduction in graying of hair, weight loss, and breathing rate were also observed in pretreated groups. Significant decline in ROS/NO and cell death in formulation pretreated mice were also observed. Decreased level of TP, LDH, and ALP in BALF/serum samples revealed G-003M-induced inhibition in lung permeability. Level of IL-6, TNF-α, and TGF-β1 in the lungs of these mice was found corresponding to control group at 8 weeks posttreatment. On the contrary, these cytokines raised significantly in 11 Gy TGR-exposed mice. Lung pneumonitis and fibrosis were found significantly countered in these mice. The observations revealed that G-003M could regulate immune system by curtailing radiation-induced oxidative and inflammatory stress, which has helped in minimizing radiation-inflicted pneumonitis and fibrosis.

## Introduction

Radiation-induced complications to the respiratory system are among one of the common causes of fatality. There are reports where radiation exposed patients were kept alive by supportive and prophylactic care but died few months later, predominantly due to pulmonary infections and pneumonitis (1). Radiation-induced pneumonitis and fibrosis in humans as well as in experimental animals have been repeatedly documented (2–5). Pneumonitis is evidenced by appearance of alveolar edema, infiltration of inflammatory cells, and extensive alveolar damage (6). Vascular injury, the early phase of an inflammatory response, mediates the release of certain pro-fibrotic cytokines such as fibroblast growth factor, transforming growth factor-β1 (TGF-β1), platelet derived growth factor (PDGF), etc. (7). Excessive inflammation accelerates collagen and extracellular matrix formation, which may end up in tissue fibrosis (5, 7). Owing to high oxygen content, production of radiation-induced reactive oxygen species (ROS)/reactive nitrogen species (RNS) is excessive in lungs. Increased ROS/nitric oxide (NO) perturbs alveolar epithelium and vascular endothelium, which may lead to recruitment of certain inflammatory cells in the lung parenchyma (5, 8). Cytokines [tumor necrosis factor-α (TNF-α), IL-1, interleukin-6 (IL-6), PDGF, fibroblast growth factor, and TGF-β1] released by inflammatory cells, consequentially lead to lung pathogenesis and subsequent loss in functional integrity of the organ (9). TGF-β1, secreted by numerous inflammatory, mesenchymal, and epithelial cells converts fibroblasts and other cell types into matrix-producing myofibroblasts finally leading to fibrosis (6, 9).

Exposure of radiation during therapies and radiological accidents (localized/whole body) has raised the need for development of countermeasures (10). Many compounds investigated in past have demonstrated limited translational potential due to their toxic nature at therapeutic doses. Amifostine (WR 2721), a synthetic sulfhydryl compound, is the only drug that has been approved by US Food and Drug Administration as a radioprotector (11). However, its use has been limited to head and neck cancer patients due to undesired signs and symptoms such as diarrhea, hypotension, hypocalcemia, and nephro- and neurotoxicity.

Lately, it was realized that natural resources due to their multi-targeted activity may be better choice for development of safe and effective countermeasures against radiation (12). Our group has also explored *Podophyllum hexandrum* derived phytomolecules (podophyllotoxin, podophyllotoxin glucoside, and rutin) against radiation exposure. Prophylactic application of our earlier formulation (G-002M), prepared by combining all the three bioactive molecules, has shown significant potential in minimizing damage to cellular biomolecules and enhanced survival in mice (13). Besides, the formulation has also revealed radioprotective effect in hepatopulmonary system of mice, predominantly by inhibiting radiation-induced ROS/NO generation (14, 15). G-002M had also shown protection to other vital organs system such as bone marrow, gastrointestinal tract, spleen, and thymus against radiation injuries (13, 16–18). Attenuation of DNA damage detected by γH2AX foci formation in human blood leukocytes, mice, and rabbit blood exposed to radiation has been revealed by G-002M (19, 20). Reduction in the number of chromosomal aberration both in *in vitro* and *in vivo* model systems further strengthen its DNA protecting potential (18, 21). The signature compound, podophyllotoxin, present in the formulation is well reported to possess properties of cell-cycle arrest at G2/M phase, regulation of DNA repair pathway, and cellular proliferation in both *in vitro* as well as *in vivo* model systems (16, 19, 21). Rutin, another compound present in the formulation, demonstrates strong proton donating and free radical stabilizing properties (16, 18). Bioinformatics studies have also strengthened the abovementioned statement (22).

The present study demonstrates the radioprotective potential of a formulation (G-003M), prepared by combining two molecules podophyllotoxin and rutin, in the lungs of thoracic-irradiated mice. To reveal the efficacy of our formulation in inhibiting radiation-induced pneumonitis and fibrosis, various assays were performed. ROS/NO inhibition demonstrated antioxidant property of G-003M. Nitrosative stress modulation was revealed by studying the expression of inducible nitric oxide synthase (iNOS) and nitrotyrosine in lung tissues. DNA protecting ability of the formulation had been demonstrated by terminal deoxynucleotidyl transferase dUTP nick-end labeling (TUNEL) assay. Anti-inflammatory potential of our formulation was evaluated by measuring infiltration of inflammatory cells in bronchoalveolar lavage fluid (BALF) [detected by cluster of differentiation 45 (CD45) immunostaining]. Histological studies, revealing anti-inflammatory potential of G-003M, signified the status of tissue injury. Reduction in radiation-induced fibrosis was confirmed by Masson’s trichrome staining/hydroxyproline (Hpl) content in lung tissues. Status of pro-inflammatory/pro-fibrogenic cytokines (IL-6, TNF-α, and TGF-β1) was measured to demonstrate immunomodulating property of G-003M. The entire data generated during the current study collectively explained that G-003M has a significant potential of minimizing radiation-induced lung fibrosis and pneumonitis.

## Materials and Methods

### Reagents and Antibodies

Goat anti-rabbit horseradish peroxidase (HRP) (sc-2030; CA, USA), anti-TNF-α (sc-52746), anti-nitrotyrosine (sc-32757), anti-CD 45 (sc-1178), and 3,3′-diaminobenzidine (DAB) substrate (7304) were procured from Santa Cruz Biotechnology Inc. (TX, USA). Polyclonal anti-TGF-β1 (AV 37894), anti-iNOS (N7782), 2′,7′-dichlorofluorescein diacetate (DCF-DA), diaminofluorescein diacetate (DAF-2), dimethyl sulfoxide (DMSO), trichloroacetic acid (TCA), chloramine-T, sodium acetate, isopropanol, *p*-dimethylaminobenzaldehyde, perchloric acid, trans-4-hydroxy-l-proline standard, and all other required chemicals were obtained from Sigma Aldrich (St. Louis, MO, USA). Mayer’s hematoxylin and eosin (H&E) stain was purchased from Fisher Scientific (Pittsburgh, PA, USA).

### Animals and γ-Ray Irradiation

Female C57BL/6 mice, an established model for studying radiation-induced pulmonary injuries (23), were selected for the current study. Mice (24 ± 2 g), aged 8–10 weeks, taken from inbred colony (INMAS, Delhi, India), were used as per the protocols approved by Institutional Animal Ethics Committee (INM/IAEC/16/21), INMAS. Animals, maintained at controlled light and temperature conditions, housed in polypropylene cages containing sterile paddy husk were fed with standard food pellet (Amrut Laboratory Animal Feed, Maharashtra, India) and water *ad libitum*. Anesthetized mice were exposed to thoracic irradiation (11 Gy) by 60Co gamma irradiator (Cobalt Teletherapy Bhabhatron-II, Mumbai, India), in specific holder designed to immobilize the animals. Throughout the study, radiation dose was maintained at 0.83 Gy/min. Dosimetry was carried out with Fricke’s chemical dosimetry method by institutional radiation safety officers.

### Experimental Design and Preparation of Radioprotective Formulation

Randomly selected healthy animals were divided in four groups (six mice per group): non-irradiated control, G-003M only treated, 11 Gy irradiated and G-003M + 11 Gy. Group G-003M + 11 Gy was administered with G-003M intramuscularly (5 mg/kg body weight of animal), −1 h to 11 Gy thoracic gamma radiation (TGR). Experiments were repeated twice.

The formulation used in the current study, coded as G-003M, is a combination of two bioactive molecules podophyllotoxin and rutin purchased from Sigma Aldrich (St. Louis, MO, USA) in their 98% purity. To prepare G-003M freshly, both the compounds were dissolved in DMSO, which was 10% of the total injectable volume (20 µl in 200 µl of total injectable). Their molecular weight and chemical structure have already been revealed in a publication by Dutta et al. (13).

### Survival and Symptomatic Monitoring of Experimental Mice

A separate group of six mice was used for survival and symptomatic monitoring studies. The animals were monitored daily for body weight, change in hair color, and survival. Survival was reported as percentage of animals surviving till 16 weeks after irradiation. The ratio of lung weight to body weight was used as lung weight coefficient (lung weight/body weight × 1,000). Mice from each treatment group were monitored for their breathing rate by using SA II (Model 1030, Small Animal Instruments, Inc., NY, USA) as per the manufacturer’s instructions. Breathing rate of six mice per group was recorded at 8 and 16 weeks posttreatment in conscious mice in relatively calm position to avoid experimental error.

### Extraction of BALF and Cellular Counts

After tracheotomy, bronchoalveolar lavage was performed in sacrificed animals using 200 µl of warmed sterile phosphate-buffered saline (PBS). The solution was instilled five times into the trachea, and fluid was withdrawn gently (24). The aspirated fluids were pooled, immersed immediately in slurry of ice and centrifuged for 10 min at 2,000 rpm. The supernatants were separated into aliquots and kept frozen at −80°C until analysis. BALF cells were resuspended in 1 ml PBS and quantified with a hemocytometer by the conventional method. Out of the total BALF cells, number of macrophages was calculated based upon their morphological identification such as large size, eccentric nuclei, and abundant cytoplasm. BALF was centrifuged; smeared on clean glass slides, stained with May-Grünwald–Giemsa and observed in blind manner under the microscope to confirm the ratio of macrophages and leukocytes, counted in hemocytometer.

### Total Protein (TP), Lactate Dehydrogenase (LDH), and Alkaline Phosphatase (ALP) Activity in BALF/Serum

Total protein, LDH, and ALP activity was measured in serum/BALF samples of experimental animals. BALF was collected from sacrificed mice at different time intervals after treatment. For serum analysis, blood collected in plain vials was centrifugated at 5,000 rpm for 10 min at 4°C. Separated serum was stored at −20°C until analysis. The level of TP, LDH, and ALP was measured in all the experimental mice by using fully automatic Biochemistry Analyzer (Erba; Model No: EM-360).

### IL-6 and TNF-α Concentrations in BALF

The IL-6 and TNF-α contents were detected in BALF of differentially treated mice by flow based Kits BDTM Cytometric Bead array (CBA) Flex Set (BD Biosciences, USA) according to the manufacturer’s instructions.

### Measurement of ROS/NO Induced by γ Radiation

Intracellular ROS/NO generation was measured in BALF cells by flow cytometry and fluorescence microscopy, 1 h posttreatment. ROS were detected using ROS-sensitive fluorescent dye DCF-DA (Sigma Co., St. Louis, MO, USA). The cells (2 × 10^4^/ml), washed with PBS, were incubated for 15 min with DCF-DA (10 µM) dye. Generation of ROS was studied using flow cytometer (BD Accuri™ C6, Becton Dickinson Biosciences, USA) and imaged with fluorescent microscope (Model No. BX-63, Olympus, Japan). To study NO formation, the cells were stained with NO-sensitive fluorescent dye 4,5-DAF-2. Data were analyzed with Image J software, and photographs were processed using Adobe Photoshop software (San Jose, CA, USA).

### Western Blotting

Proteins isolated from the frozen lung tissues were quantified by Bradford method (25). Denatured protein was subjected to 12% SDS-PAGE and transferred to Whatman PROTRAN nitrocellulose transfer membrane (Sigma). After blocking the non-specific sites with 5% (w/v) skimmed milk for 2 h, the membranes were incubated overnight at 4°C with primary antibodies anti-TGF-β1 (1:1,000)/iNOS (1:1,000)/nitrotyrosine (1:1,000). The membranes were then incubated with HRP-conjugated goat anti-rabbit IgG (1:5,000) for 2 h at room temperature. Immunoreactive bands were visualized using an enhanced chemiluminescence detection system. Densitometry was performed on the resulting autoradiograph using Image Lab software of BioRad Gel Documentation System (Gel Doc XR; Cat. No. 1708195).

### Lung Weight Coefficient and Histology

Whole lung was excised from animals dissected at different time intervals. Lung tissues, individually from all the mice, were weighed after blotting. Lung weight coefficient was calculated by using the formula (lung weight/body weight × 1,000). For histological and immunohistochemical studies, lung tissues were fixed in 10% formalin. In separate groups of animals, whole lung was used for determining Hpl content.

### Histological Analysis and Fibrosis Estimation Using Masson’s Trichrome Staining

Differentially treated mice from all the groups were dissected at different time intervals following treatment. The lung tissues, excised individually from each mouse, were fixed in 10% formalin solution and dehydrated in series of alcohol. Paraffin-embedded tissues were sectioned at thickness of 5 µm with a semiautomatic microtome (Spencers, Gurgaon, India). The slides were stained with H&E to evaluate architectural changes in lung tissues. The extent of damage was assessed by Eldh et al. method (26). Images were obtained by using digital camera mounted on BX-63, Olympus microscope. Quantification of damage was performed by scoring from 0 to 4 based on the amount of area affected by interstitial inflammation, alveolar wall thickening, and peribronchial inflammation (26).

The sections stained with Masson’s trichrome were subjected to Ashcroft score for evaluating lung fibrosis (27). Severity of the fibrosis was graded and scored on a scale of 0–8. Ten fields per sections from the individual mice were randomly selected and scored at 400× magnification. Assessment was performed in double-blind fashion. After whole section examination, fibrosis score was evaluated as mean score of all the fields.

### Lung Hpl Content

Hydroxyproline content in lung tissues of all the differentially treated mice was determined by colorimetric assay (28). Briefly, individual tissue was homogenized with 2 ml distilled water and incubated with 125 µl of 50% TCA on ice for 20 min. After centrifugation, the pellet was mixed with 1 ml hydrochloric acid (2 N) and baked at 110°C for 14–18 h until charred and dry. The acid dried samples were then re-suspended in 2 ml deionized water and mixed with 500 µl 1.4% chloramine-T in 0.5 M sodium acetate/10% isopropanol. After 20 min incubation at room temperature, 500 µl of Ehrlich’s solution (1.0 M *p*-dimethylaminobenzaldehyde in 70% isopropanol/30% perchloric acid) was added, mixed and incubated at 65°C for 15 min. Absorbance of each individual sample was measured at 550 nm. Concentration of lung Hpl was calculated by Hpl standard curve prepared by serial dilutions of trans-4-hydroxy-l-proline standard (Sigma, St. Louis, MO, USA). Samples were assayed in triplicate.

### Detection of Cell Death Using TUNEL Assay

Cell death was detected by the TUNEL method using TUNEL apoptosis detection kit (catalog no. 17-141; Upstate Technology, Lake Placid, NY, USA). Briefly, 5 µm sections were deparaffinized, dehydrated through series of alcohol and incubated with proteinase K [1/24 (v/v) in PBS] for 15–30 min at 37°C. After stopping proteinase K digestion reaction with PBS, the samples were incubated with terminal deoxynucleotidyl transferase end-labeling cocktail (a mixture of terminal deoxynucleotidyl transferase buffer, biotin-dUTP, and terminal deoxynucleotidyl transferase in ratio of 90:5:5, respectively) for 60 min at 37°C. Following washing and blocking, avidin–fluorescein isothiocyanate (1:10) was applied to the samples and incubated in dark for 30 min at 37°C. The slides were washed with PBS, counterstained with propidium iodide, and visualized under fluorescent microscope (Model No. BX-63, Olympus, Japan). Dead cells were quantified by counting the TUNEL+ cells using pseudo-colored overlay images prepared by ImageJ software (National Institutes of Health, Bethesda, MD, USA). Percentage of TUNEL+ cells was obtained against the total number of nucleated cells (propidium iodide counter stained) in 10 different fields per tissue section.

### Immunohistochemical Studies

Lung tissue sections were processed to evaluate immunohistochemical expression of CD45, TGF-β1, TNF-α, iNOS, and nitrotyrosine. Deparaffinized sections were passed through graded series of ethanol (100, 90, 70, and 50%), and slides were washed in distilled water. Subsequently, the sections were incubated with 0.3% H_2_O_2_ for 30 min to block endogenous peroxidase activity before performing heat-induced antigen retrieval. After antigen retrieval in sodium citrate buffer (10 mM sodium citrate, 0.05% Tween 20, pH 6.0), slides were washed, and expression of antigens was detected by applying DAB substrate solution. The sections were counterstained with hematoxylin, dehydrated, mounted, and scored for immunopositive cells under a light microscope. Inflammatory infiltrates were evaluated by counting CD45+ aggregates in 10 randomly selected fields, per lung tissue section. Results were obtained by scoring the sections blindly by at least two individuals.

### Statistics Analysis

Statistical tool SPSS (Statistical Package for Social Science; Windows version 10.0) packed program was used for finding significance values of data. Values were expressed as mean values ± SEM and for analysis of differences between the groups. One-way ANOVA was used with Newman–Keuls *post hoc* test. Statistical differences are presented at probability levels of *p* < 0.05, <0.01, and <0.001.

## Results

### G-003M Improves Survival and Other Symptomatic Features

**Figure 1:**
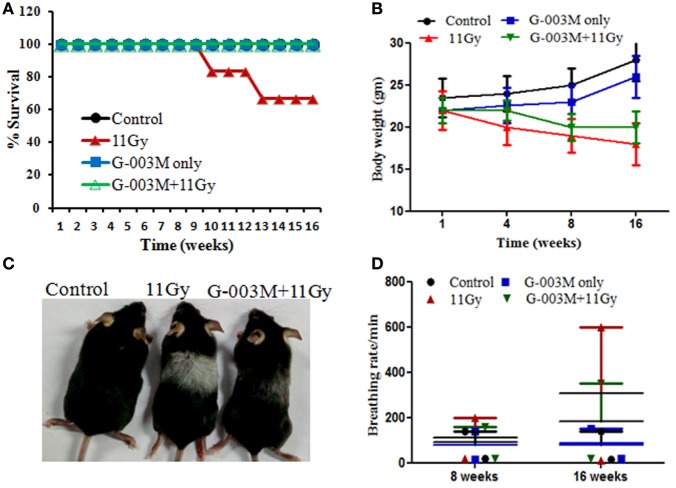
**(A)** Percent survival of female C57BL/6 mice (8–10 weeks old) exposed to thoracic gamma radiation (11 Gy) and pretreated with G-003M formulation. Survival was recorded up to 16 weeks postirradiation (*n* = 6 for each group). **(B)** Body weight was recorded at different time intervals after experimentation. Data are reported as means ± SEM from six mice for each experimental group. **(C)** Pattern of change in hair color of mice in irradiated thoracic region. **(D)** Effect of G-003M on breathing rate of 11 Gy thoracic-irradiated mice. Breathing rate of individual mice was measured at 8 and 16 weeks posttreatment. Data represent the average breathing rate value obtained from six mice from each experimental group.

Radiation-induced pneumonopathy is associated with body weight loss and compromised survival. Therefore, we evaluated the effect of G-003M on survival, body weight, and other symptomatic features of irradiated mice. Percent survival in thoracic-irradiated C57BL/6 mice was measured in all the experimental groups. In radiation only group, all the animals survived up to 8 weeks postirradiation; however, in 10 weeks survival reduced to 83.33%. Survival in this group further dropped to 66.50% by 14 weeks (Figure 1A). In G-003M pretreated groups, all the animals survived up to the last time point of the study (16 weeks of experimentation) (Figure 1A). The body weight of control and G-003M only treated mice increased in natural way with time. However, in TGR treated group, weight loss was insignificant in first 4 weeks of radiation exposure. A remarkable fall (18–20%) in body weight was recorded within 8–16 weeks in this group (Figure 1B). In G-003M pretreated group, weight loss was much less (8–10%; *p* < 0.001) in comparison to the corresponding radiation-treated group (Figure 1B). After 8 weeks of irradiation, hair of the C57BL/6 mice turned gray in the thoracic region, the area of exposure (Figure 1C). In irradiated group, this gray color band was extremely apparent in comparison to G-003M treated mice. G-003M only treatment exhibited same hair color as in control group.

### G-003M Mitigates Increased Breathing Rate in Irradiated Mice

Radiation manifests lung injury in the form of acute pneumonitis and fibrosis. Development of fibrosis in lungs usually results in difficult breathing and increase in breathing rate in experimental animals as well as in humans (29, 30). In this study, increased breathing rate, yet not significant, was observed in irradiated animals by 8 weeks of radiation exposure, and it was markedly higher (fourfold; *p* < 0.001) at 16 weeks in comparison to respective untreated controls of the same age (Figure 1D). However, in G-003M-administered mice, breathing rate was much less (*p* < 0.001; G-003M + 11 Gy vs. 11 Gy) when compared with irradiated mice at 16 weeks of experimentation (Figure 1D). Treatment of G-003M only exhibited normal breathing rate in mice at all the time intervals.

### G-003M Reduces Generation of ROS, NOS, and Nitrosative Stress

**Figure 2:**
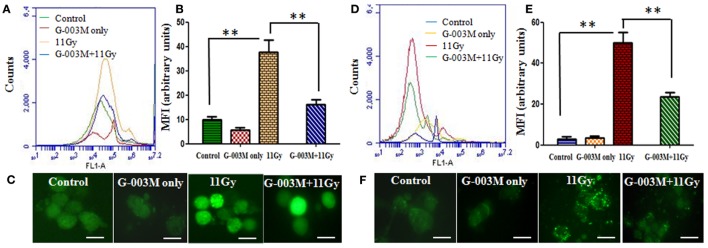
Effect of G-003M on reactive oxygen species (ROS)/nitric oxide (NO) formation in bronchoalveolar lavage cells of 11 Gy thoracic-irradiated mice. **(A)** Histogram representing changes in ROS formation in bronchoalveolar lavage fluid (BALF) cells detected by ROS-sensitive fluorescent dye 2′,7′-dichlorofluorescein diacetate, 1 h posttreatment. **(B)** Bar graph indicating the mean fluorescence intensity (MFI) detected at a 488 nm excitation and a 535 nm emission using flow cytometer. **(C)** Fluorescence microscopic images of radiation-induced ROS formation in BAL cells from the lungs of differentially treated mice. **(D)** Histogram representing NO generation in BALF cells at 1 h posttreatment. NO was detected by staining the cells with NO-sensitive fluorescent dye 4,5-diaminofluorescein diacetate. **(E)** Bar graph indicating the MFI. **(F)** Fluorescence microscopic images of NO generation in BAL cells. Images were viewed at 1,000× under fluorescence microscope. Scale bar 10 µm. Data are represented as means ± SEM from six mice for each group. **p < 0.001.

Radiation induces damage predominantly by generation of ROS and NO in cellular milieu. In the current study, formation of ROS and NO was measured in BALF cells of experimental mice at 1 h post-TGR exposure. As evident in Figures 2A–C, ROS formation was found to be markedly (*p* < 0.001) elevated in BALF cells of TGR-exposed mice in comparison to controls. NO production, measured by using fluorescent dye 4,5-DAF-2, was also prominently raised in irradiated group (Figures 2D,F). However, pre-irradiation administration of G-003M significantly (*p* < 0.001) countered both ROS and NO production in BALF cells of irradiated mice. In G-003M only treated group no ROS and NO formation was evident at 1 h after treatment.

**Figure 3:**
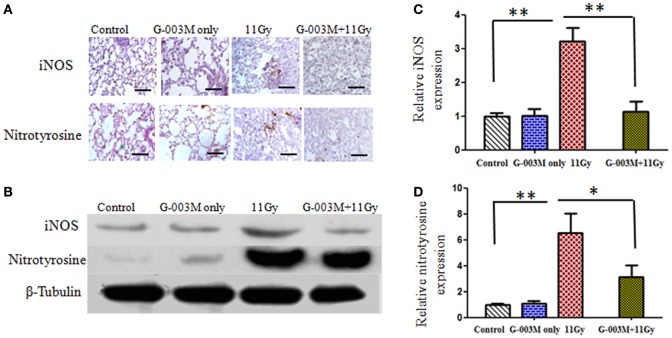
Effect of G-003M on nitrosative stress in 11 Gy thoracic-irradiated lungs. **(A)** Immunohistochemical expression of inducible nitric oxide synthase (iNOS) and nitrotyrosine in lungs of differentially treated mice (200×). **(B)** Western blotting of iNOS and nitrotyrosine in lungs at 8 weeks postirradiation treatment. Densitometry analysis represents iNOS **(C)** and nitrotyrosine **(D)** expression normalized to β-tubulin. Scale bar 100 µm. Data are represented as means ± SEM from six mice for each group. *p < 0.01, **p < 0.001.

Radiation-induced nitrosative stress was assessed by expression of iNOS and nitrotyrosine in lungs of experimental mice. Peroxynitrite (ONOO^−^), a RNS, is detected in the tissues as nitrotyrosine, which is a stable product formed by reaction of ONOO^−^ with tyrosine. Figures 3A,B show iNOS and nitrotyrosine expression measured by immunohistochemistry and immunoblotting techniques in different treatment groups. Immunohistochemical study revealed that both iNOS and nitrotyrosine staining was not evident in lungs of control mice but significantly apparent in irradiated animals (Figure 3A). iNOS expression increased from first week and maximum at 8 weeks of posttreatment. Nitrotyrosine staining was intense in alveolar macrophages and at the site of injury (Figure 3A). Immunoblotting of iNOS and nitrotyrosine in lung tissues further validate these findings. Intense band of iNOS and nitrotyrosine was detected in irradiated group in comparison to controls (Figures 3B–D). However, iNOS and nitrotyrosine expression was not as marked as that of irradiated groups with the pretreatment of G-003M (Figures 3A,B). Expression of iNOS and nitrotyrosine was similar to controls in animals that were treated with G-003M only.

### G-003M Inhibits Radiation-Induced Cell Death of Pulmonary Cells

**Figure 4:**
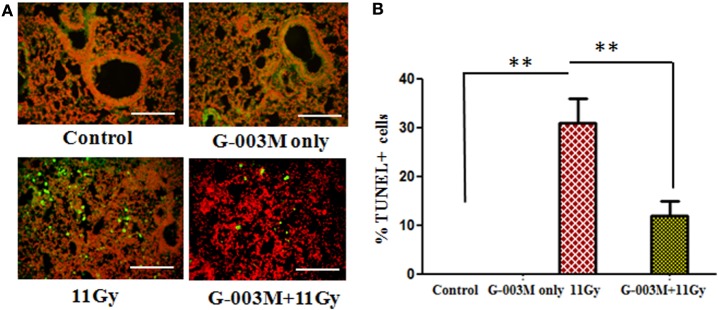
G-003M protects pulmonary cells from radiation-induced cell death in 11 Gy thoracic-irradiated mice. DNA fragmentation was performed in lung sections using *in situ* terminal deoxynucleotidyl transferase dUTP nick-end labeling (TUNEL) method. **(A)** Representative micrographs of TUNEL (green) and PI (red) double staining in differentially treated mice at 1 week postradiation. **(B)** Bar graph showing the average number of TUNEL+ cells in lungs. Images were viewed at 200× under fluorescence microscope. Scale bar 100 µm. Data are represented as means ± SEM from six mice for each group. **p < 0.001.

Free radicals produced by radiation play an important causative role in cell death. In the current investigation, cell death was detected in lung tissues by using TUNEL assay. As evident in Figures 4A,B, remarkable increase in the number of dead (TUNEL+) pulmonary cells was observed in 11 Gy thoracic-irradiated group as compared to untreated controls. Administration of G-003M formulation, 1 h prior to radiation, indicated significant (*p* < 0.001) reduction in the number of TUNEL+ pulmonary cells showing efficacious function of formulation against radiation-induced cell death (Figures 4A,B). No TUNEL+ cells were observed in control and G-003M only treatment group.

### G-003M Modulates Depletion of Total BALF Cellular Counts Including Macrophages

**Figure 5:**
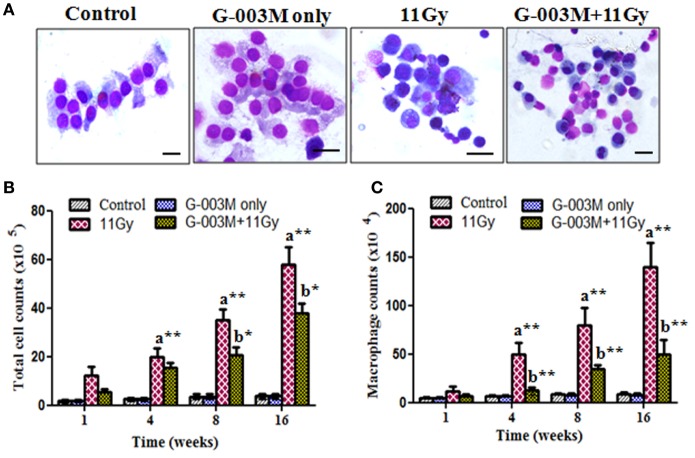
Effect of G-003M on bronchoalveolar lavage fluid (BALF) of irradiated mice. **(A)** Representative images of May-Grünwald–Giemsa stained cells from BALF of differentially treated mice. Control and G-003M only groups showing normal epithelial cells and macrophages. Enhanced number of inflammatory cells in BALF of 11 Gy thoracic-irradiated mice. Noticeable reduction in the number of these inflammatory cells in G-003M-pretreated irradiated group. Magnification 1,000×. **(B)** Total BALF cellular counts and **(C)** macrophages counts in BALF. Scale bar 10 µm. Data are represented as means ± SEM from six mice for each group. a = 11 Gy vs. controls, b = G-003M + 11 Gy vs. 11 Gy. *p < 0.05; **p < 0.001.

Recruitment of inflammatory cells into the lung has been associated with radiation-induced pneumonitis. Anti-inflammatory effect of G-003M formulation was evaluated by measuring BALF cells in lungs of irradiated mice. Nearly sixfold increase was seen in BALF cellular counts at 1 week of radiation exposure (Figures 5A–C). The number of these inflammatory cells amplified further and was maximum (about 14-fold increase; *p* < 0.001, 11 Gy vs. controls) at 16 weeks of study in this group. When number of macrophages was calculated, marked (*p* < 0.001) increase was observed from 4 to 16 weeks in irradiated group as compared to their respective controls. Interestingly, G-003M-pretreated mice showed significantly less number of macrophages and other BALF cells at all the time intervals of study when compared with irradiated groups. However, values were still higher in comparison to respective controls (Figures 5B,C). The number of BALF cells was found similar to controls in G-003M only treatment group at all the time intervals.

### G-003M Reduces Radiation-Induced Lung Permeability and Cell Damage

Radiation induces endothelial injury resulting in increased vascular permeability in lungs. Lung permeability and cellular damage were assessed in the current study by evaluating TP content, LDH, and ALP activity in serum/BALF of experimental mice. TGR resulted markedly enhanced (*p* < 0.001) level of proteins in BALF of irradiated groups in the first week of treatment (Table 1). The level of protein continued to rise significantly in BALF of irradiated animals with the advancement of time. No change in protein level was recorded in serum samples of irradiated animals. TGR-exposed mice showed nearly 1.5-fold increase in LDH activity in serum and BALF samples even in first week of treatment (Table 1). The level of LDH was found to increase in this group up to 16 weeks of study in both serum and BALF samples. No significant increase in ALP activity was observed within 4 weeks in BALF of irradiated animals. However, the activity increased significantly (*p* < 0.001) at 8 weeks and found to be very high (twofold increase) at 16 weeks of study in this group. Pretreatment of G-003M significantly declined the level of TP, LDH, and ALP in BALF/serum of irradiated animals at all the time points of study. However, values of protein, LDH, and ALP in BALF of irradiated animals were higher in comparison to respective controls (Table 1). G-003M only treatment exhibited no significant changes in the level of TP, LDH, and ALP in BALF/serum of mice at any studied time interval.

**Table 1:** Level of lactate dehydrogenase (LDH), total protein (TP), and alkaline phosphatase (ALP) in serum/bronchoalveolar lavage fluid (BALF) of differentially treated C57BL/6 mice.

Time intervals	Groups	Serum		BALF		
		LDH (U/l)	TP (g/dl)	LDH (U/l)	TP (g/dl)	ALP (U/l)
1 week	Control	547.53 ± 15.23	2.52 ± 0.21	102.33 ± 23.56	0.00 ± 0.00	18.51 ± 3.30
	G-003M only	458.42 ± 12.63	2.58 ± 0.23	113.47 ± 25.69	0.04 ± 0.00	15.60 ± 2.55
	11 Gy	1,211.35 ± 11.56^a^**	2.59 ± 0.26	218.95 ± 20.25^a^**	0.30 ± 0.02^a^**	20.00 ± 3.56
	G-003M + 11 Gy	727.23 ± 31.37^b^**	2.84 ± 0.28	115.80 ± 15.36^b^*	0.13 ± 0.01^b^**	15.00 ± 2.35

4 weeks	Control	560.21 ± 17.56	5.42 ± 0.45	109.56 ± 15.36	0.03 ± 0.00	23.50 ± 3.50
	G-003M only	489.10 ± 12.25	5.46 ± 0.42	125.64 ± 12.57	0.02 ± 0.00	28.52 ± 2.50
	11 Gy	703.10 ± 19.25^a^*	5.25 ± 0.48	249.00 ± 21.32^a^**	0.35 ± 0.03^a^**	29.00 ± 3.60
	G-003M + 11 Gy	605.00 ± 21.20^b^*	5.56 ± 0.38	233.00 ± 20.12	0.20 ± 0.01^b^*	11.00 ± 2.30

8 weeks	Control	603.23 ± 10.25	5.89 ± 0.47	125.25 ± 12.56	0.09 ± 0.00	28.36 ± 3.50
	G-003M only	509.30 ± 12.15	5.26 ± 0.43	168.15 ± 10.78	0.04 ± 0.00	25.51 ± 2.32
	11 Gy	897.80 ± 12.36^a^**	5.55 ± 0.54	1,194.66 ± 312.74^a^**	0.40 ± 0.05^a^**	50.00 ± 5.23^a^**
	G-003M + 11 Gy	614.00 ± 15.63^b^**	5.69 ± 0.39	302.5 ± 38.5^b^**	0.23 ± 0.01^b^**	40.00 ± 4.50^b^*

16 weeks	Control	861.00 ± 129.13	5.72 ± 0.04	403.52 ± 110.92	0.12 ± 0.06	30.39 ± 2.30
	G-003M only	745.00 ± 131.23	5.66 ± 0.34	512.69 ± 99.65	0.10 ± 0.05	28.56 ± 3.27
	11 Gy	2,375.50 ± 218.77^a^**	5.72 ± 0.39	1,278.56 ± 177.48^a^**	0.42 ± 0.04^a^**	65.00 ± 5.45^a^**
	G-003M + 11 Gy	1,162.50 ± 115.82^b^**	5.43 ± 0.31	765.23 ± 125.82^b^**	0.26 ± 0.02^b^**	45.00 ± 7.25^b^*

*Values are expressed as mean ± SEM of serum and BALF collected individually from all the groups of experimental animals. Experiments were performed in duplicate having six animals in each group*.

*^a^11 Gy vs. control*.

*^b^G-003M + 11 Gy vs. 11 Gy*.

*p < 0.01.

**p < 0.001.

### G-003M Attenuates Radiation-Induced Changes in Lung Architecture and Fibrosis

**Figure 6:**
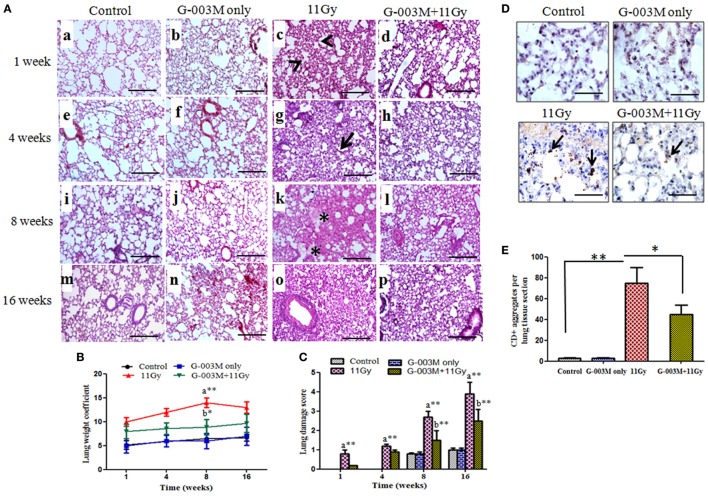
Effect of G-003M on lung inflammation and injury of 11 Gy thoracic-irradiated mice. **(A)** Representative light micrographs showing changes in lung histology at different time intervals. The alveoli in control group were normal on week 1 (a), 4 (e), 8 (i), and 16 (m). Irradiated lungs showing infiltration of inflammatory cells (c, arrow heads) and thickened alveolar septa (g, arrow) on 1 and 4 weeks. On week 8, alveolar edema (*) was visible and alveolar space was reduced by collagen deposition (k). Complete distortion of lung architecture and alveolar collapse on 16 weeks (o). In G-003M-pretreated groups inflammation, extent of lung damage and fibrosis was significantly less in comparison to irradiated groups on week 1 (d), 4 (h), 8 (l), and 16 (p). Lung sections were stained with hematoxylin and eosin and studied under light microscope (200×). Scale bar 100 µm. **(B)** Bar graph of lung weight coefficient from differentially treated groups. **(C)** Semiquantitative analysis of lung damage score. **(D)** Immunohistochemical staining of CD45+ leukocytes in lung of differentially treated mice at 4 weeks after treatment (1,000×). Representative photomicrograph from irradiated lungs shows intense CD45+ aggregates (arrows), indicating infiltration of inflammatory cells. G-003M-pretreated group representing less number of CD45+ cell aggregates in comparison to irradiated group. Control and G-003M only groups showing very few CD45+ cells in lung sections. Scale bar 10 µm. **(E)** Quantification of lung CD45+ inflammatory aggregates per section (10 fields) of individual mice identified by IHC staining. Data are represented as means ± SEM from six mice for each group. a = 11 Gy vs. controls, b = G-003M + 11 Gy vs. 11 Gy. *p < 0.05; **p < 0.001.

Pulmonary inflammation and fibrosis were estimated by histological studies. H&E and Masson’s trichrome-stained slides were observed for radiation-induced inflammatory cells infiltration in pulmonary alveoli and appearance of fibrosis at later phase. Infiltration of inflammatory cells and alveolar wall thickening was observed in lungs of irradiated mice from 1 to 8 weeks postirradiation (Figure 6A, c,g). Congestion of the alveoli, presence of foamy macrophages around the bronchioles and severe interstitial edema was recorded in the lungs from 4 to 8 weeks of postirradiation (Figure 6A, k). Lung edema measured by calculating lung weight coefficient was found maximum at 8 weeks postirradiation treatment (Figure 6B). At later phase (8–16 weeks), acute pneumonitis was followed by marked fibrotic changes as evident in H&E stained sections (Figure 6, o). Lung damage score was maximum in irradiated animals at 16 weeks postexposure (Figure 6C). However, pre-administration of G-003M showed significantly less interstitial edema, infiltration of inflammatory cells, and alveolar wall thickening in irradiated lungs (Figure 6, d,h,l,p). The formulation significantly (*p* < 0.01) lowered the values of lung weight coefficient in irradiated mice at all the time intervals, indirectly indicating reduction in radiation-induced pneumonedema. Lung damage score was also significantly minimized by G-003M pretreatment (Figure 6C). However, lung architecture was not completely normalized in this group in the study period. No pathological alteration in lung architecture was observed in the animals of G-003M only group.

To confirm infiltration of inflammatory cells in lungs after radiation exposure, immunostaining for common leukocyte marker CD45 was applied. Radiation caused a prominent increase (*p* < 0.001; 11 Gy vs. controls) in CD45+ cell aggregates in lungs of mice by 4–8 weeks after exposure (Figures 6D,E). These cells were predominantly distributed around the vesicular vessels and also found scattered in alveolar spaces. Significantly (*p* < 0.05) low number of CD45+ cell aggregates in the lungs of G-003M-pretreated mice indicated less inflammation in comparison to irradiated (Figures 6D,E). CD45-staining intensity in G-003M only group was similar to controls at all the time intervals.

**Figure 7:**
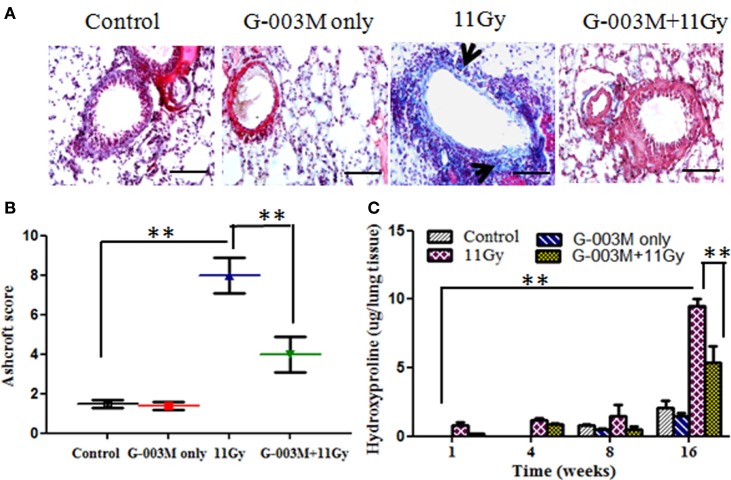
Effect of G-003M on radiation-induced fibrosis in 11 Gy thoracic-irradiated mice. **(A)** Photomicrographs of Masson’s trichrome-stained lung sections from differentially treated experimental groups at 16 weeks posttreatment. No significant fibrosis was found in the lungs of control and G-003M only treatment groups. About 11 Gy thoracic-irradiated lungs sections showed intense collagen fibers dyed blue (arrows). Compared to irradiated group, collagen deposition/fibrosis was significantly less in G-003M-pretreated irradiated group. Magnification 400×. **(B)** Evaluation of lung fibrosis by Ashcroft score at 16 weeks posttreatment. **(C)** Quantitative analysis of hydroxyproline content in lungs of experimental mice. Scale bar 50 µm. Data are represented as means ± SEM from six mice for each group. **p < 0.001.

Lung fibrosis was also studied by histological grading of fibrosis according to the criteria of Ashcroft et al. (27), using Masson’s trichrome-staining method (Figures 7A,B). Extensive deposition of collagen, as evident by light blue staining of collagen fibers around vessels and bronchioles, was observed in lungs of irradiated mice (Figure 7A). However, G-003M significantly attenuated the deposition of collagen in lungs of irradiated mice indicating its anti-fibrotic potential (Figure 7A).

Hydroxyproline content, the major constituent of collagen, was also measured in the lungs of experimental mice. No significant difference was recorded in Hpl content up to 8 weeks after radiation treatment in comparison to controls (Figure 7C). However, Hpl content was found remarkably increased (about 10-fold) at 16 weeks of TGR exposure. G-003M pretreatment had significantly (*p* < 0.001) lowered Hpl content in lungs of irradiated animals (Figure 7C). As a whole, the results indicated anti-inflammatory and anti-fibrotic effects of G-003M formulation in lungs of irradiated mice. Hpl content was similar to controls in G-003M only treatment group at all the studied time intervals.

### G-003M Reduces Expression of Radiation-Induced Inflammatory/Fibrogenic Cytokines

**Figure 8:**
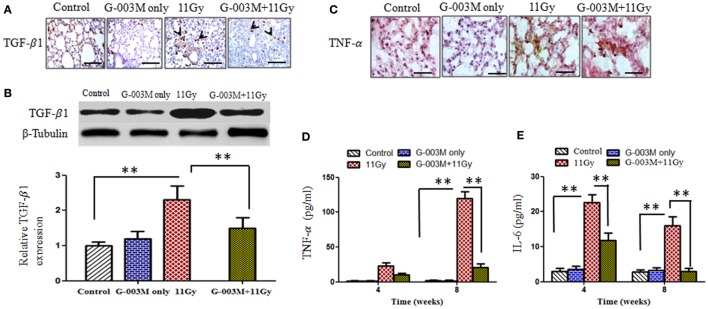
Effect of G-003M on expression of pro-inflammatory and fibrogenic cytokines. **(A)** Immunohistochemical expression of transforming growth factor-β1 (TGF-β1) (arrows) in lungs of differentially treated mice (400×). Scale bar 50 µm. **(B)** Western blotting of TGF-β1 at 8 weeks postirradiation. Densitometry analysis represents TGF-β1 expression normalized to β-tubulin. **(C)** Immunohistochemical expression of tumor necrosis factor-α (TNF-α) in lungs of experimental mice (1,000×). Scale bar 10 µm. **(D)** Histogram representing TNF-α level in bronchoalveolar lavage fluid (BALF) of experimental mice. **(E)** Bar diagram indicating interleukin-6 (IL-6) level in BALF of experimental mice. Data are represented as means ± SEM from six mice for each group. **p < 0.001.

Transforming growth factor-β1 is the key cytokine involved in progression of fibrosis. In the current study, expression of TGF-β1 was observed in lungs of mice by immunohistochemistry and further confirmed by western blotting (Figures 8A,B). Immunohistochemical staining revealed stronger expression of this cytokine in alveolar macrophages and lymphocyte of irradiated lungs as compared to controls (Figure 8A). The intensity of expression was significantly higher at 8 weeks. Figure 8B demonstrates expression of TGF-β1 by western blotting in lungs of mice sacrificed at 8 weeks postirradiation. Significant increase in band intensity was observed in irradiated lungs in comparison to controls. Pretreatment of G-003M significantly declined the expression of this cytokine in 11 Gy-irradiated lung tissues (Figure 8). However, the expression of TGF-β1 in this group was still higher than controls. No significant alteration in the expression of TGF-β1 was seen in lungs of mice treated with G-003M only.

Tumor necrosis factor-α and IL-6 are the major pro-inflammatory cytokines involved in lung inflammation. The concentration of TNF-α and IL-6 was measured in BALF of experimental mice at 4 and 8 weeks posttreatment by using flow based Kits BDTM CBA Flex Set (BD Biosciences, USA). The level of TNF-α was very high (*p* < 0.001) at 4 weeks (22.78 ± 3.50 pg/ml) and increased (119.41 ± 10.50 pg/ml) further by 8 weeks in BALF of irradiated animals when compared to controls (2.00 ± 0.10 pg/ml) (Figure 8D). TNF-α expression was also located in lung tissues by immunohistochemistry (Figure 8C). Intense expression of this cytokine was observed in irradiated lungs at 8 weeks, confirming our previous finding. However, G-003M pretreatment significantly (*p* < 0.001) reduced (10.4 ± 2.10 pg/ml at 4 weeks and 20.58 ± 3.50 pg/ml at 8 weeks) the level of TNF-α in BALF as well as its immunohistochemical expression in lungs of irradiated animals (Figures 8C,D).

Interleukin-6 concentration was also increased remarkably in BALF of irradiated animals in comparison to controls (2.80 ± 0.50 pg/ml). The level of IL-6 raised at 4 weeks (22.60 ± 2.50 pg/ml) and then declined at 8 weeks (15.96 ± 2.80 pg/ml) in this group (Figure 8E). G-003M significantly (*p* < 0.001) reduced (11.76 ± 2.2 pg/ml at 4 weeks and 2.95 ± 2.22 pg/ml at 8 weeks) the level of this inflammatory cytokine in BALF of irradiated animals (Figure 8E). No significant change in TNF-α and IL-6 was observed by G-003M only treatment in comparison to controls.

## Discussion

Toxic endpoints in the form of radiation pneumonitis and pulmonary fibrosis are frequent outcomes following radiological accidents and therapies. Radiation-mediated lung injury generally fails to fully repair and the tissue enters in dysregulated process leading to malfunctioning of the organ. Radiation exposure elicits induction of ROS and other RNS mainly NO and peroxynitrite (ONOO^−^), which further amplify tissue damage (8, 31). Since ROS/RNS are prevalent in radiation-induced lung injuries, use of antioxidant molecules/enzymes has been frequented to reduce the formation of these species in the related organ (10). Current study reveals high antioxidant potential of G-003M, documented by significant scavenging of radiation-induced ROS/NO generation (Figure 2). The study reports excessive expression of iNOS and nitrotyrosine in alveolar macrophages and epithelial cells of irradiated lungs, confirming NO formation by these cells. Decrease in iNOS and nitrotyrosine expression by G-003M administration strengthens its NO scavenging potential. The ROS and NOS scavenging property of G-003M has been achieved major by the presence of rutin in this formulation. Rutin, a bioflavonoid, contains 10 hydrogen donor counts and 16 hydrogen acceptor counts attached at different positions. The process of donating and accepting hydrogen atom stabilizes free radicals and thus reduces ROS/NO generation. These findings are in consonance with our previous report where earlier formulation (G-002M) had shown decline in ROS/NO generation in the lungs of 7 Gy whole-body-irradiated mice (14). Our current findings are also in agreement with other reports revealing amelioration in radiation-induced lung damage by exogenous intervention of agents having high antioxidant potential (29, 32–34).

Radiation is known to cause apoptosis of lung epithelial cells, which may lead to desquamation of alveolar walls and capillary luminal dilatation resulting in increased vascular permeability and interstitial edema (35). Enhanced level of alveolar protein indicates increase in vascular permeability as direct injury by radiation (35). Similar to these reports, current study also reveals excessive proteins in BALF of irradiated mice indicating radiation-induced lung injury (Table 1). Various earlier studies have also demonstrated damage to lung parenchymal cells by increased levels of LDH and ALP (36). ALP activity in BALF has been known to be associated with damage of pneumocyte type II cell, playing important role in repair of damaged alveolar epithelial cells (37). Current study also reports elevated level of TP, LDH, and ALP in BALF/serum samples of 11 Gy-irradiated animals, which has been significantly ameliorated by G-003M pre-administration (Table 1). Our formulation could also minimize cell death in irradiated lungs and maintained cellular integrity and vascular permeability leading to retention of TP, LDH, and ALP activity in BALF.

Histological findings and CD45 immunostaining in the current study have demonstrated significant reduction in radiation-induced inflammation by G-003M pre-administration. Enhanced inflammation has been documented as one of the possible mechanism of lung damage (5–7). Mechanistically, injured epithelial and other inflammatory cells produce a variety of cytokines and chemokines, which amplify inflammatory response and trigger fibroblast proliferation in irradiated lungs (38). TNF-α is an important cytokine known to trigger the production of other pro-inflammatory cytokines (39). Increased IL-6 has been reported for causing pneumonitis in post-thoracic radiotherapy patients (40). In the current study, radiation-induced overexpression of TNF-α and IL-6 in the lungs was found significantly curtailed by G-003M intervention, which had helped in reducing radiation-induced pulmonary inflammation.

Transforming growth factor-β1, a pleiotropic growth factor, plays pivotal role in pulmonary fibrosis by promoting activation, proliferation, and differentiation of epithelial cells and collagen-producing myofibroblasts (41). TGF-β1 also promotes a variety of pro-inflammatory and fibrogenic cytokines such as TNF-α, IL-1β, and IL-13. This increase further perpetuates the fibrotic cascade (42). We have currently reported, G-003M mediated significant decline in the expression of TGF-β1 which otherwise could have culminated into lung fibrosis at late phase (Figures 8A,B). The declined accumulation of collagen shown by Masson’s trichrome staining (Figure 7A) and Hpl content (Figure 7C) in formulation-pretreated mice has also supported anti-fibrotic role of G-003M. In consonance, there are several reports stating the use of lignans and other polyphenols for reducing radiation-induced lung damage by countering inflammation and inhibiting pro-inflammatory and fibrogenic cytokines like IL-6, TNF-α, and TGF-β1 (34). Vujaskovic et al. have reported that amifostine mediated radioprotection to lungs by plummeting ROS and macrophages accumulation and decreasing the expression of TGF-β1 in irradiated lung tissues (43) of rats.

Radiation-induced acute inflammatory pneumonitis and late fibrosis are known to affect breathing rate (44). In the current study also, 11 Gy thoracic irradiation caused significant increase in breathing rate notably at 16 weeks postexposure possibly due to reduced air clearance rate by fibroid lungs. Breathing rate in G-003M-pretreated animals was comparatively less affected due to reduced inflammation and minimal fibrosis formation. This activity of our formulation assisted in prolonged survival of mice irradiated locally to thoracic region. These findings are in agreement with a report in which soy isoflavones modulated breathing rate in 12 Gy thoracic-irradiated mice, confirming its radioprotective efficacy (45).

**Figure 9:**
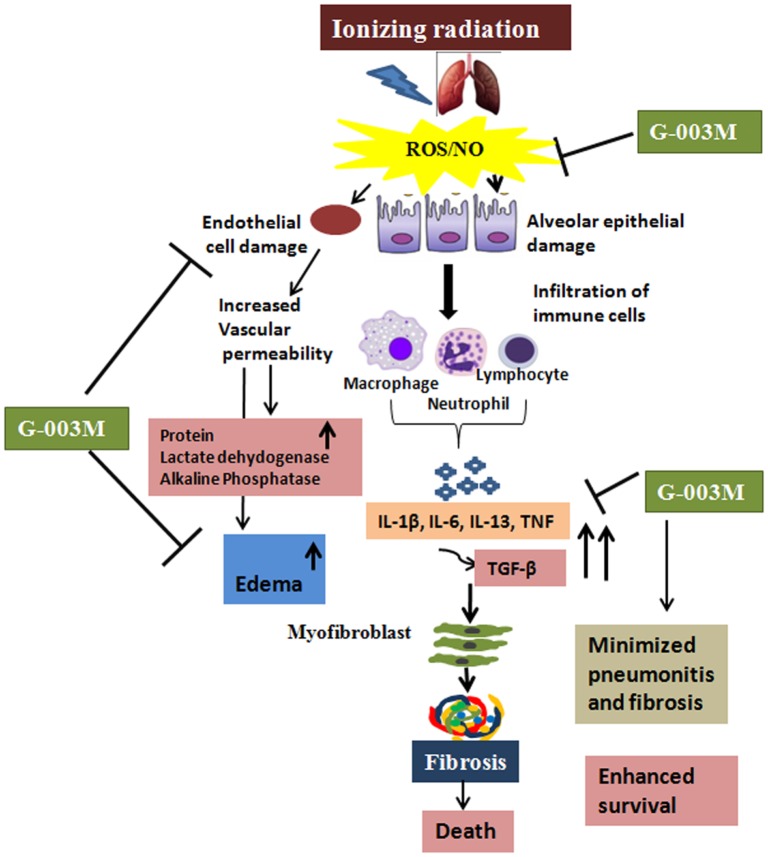
Diagrammatic illustration showing the possible mechanisms by which G-003M is reducing radiation-mediated pneumonitis and pulmonary fibrosis. Thoracic exposure to radiation results in rapid production of reactive oxygen species (ROS)/nitric oxide (NO), leading to epithelial and endothelial cell damage followed by extensive infiltration of the immune cells. Further increase in the pro-inflammatory and pro-fibrogenic cytokines leads to activation of myofibroblast, thus causing fibrosis and death. Administration of G-003M formulation stabilizes the highly reactive free radicals (ROS and NO), thus preventing the epithelial and endothelial cell damage and further infiltration of the immune cells. G-003M promotes survival against radiation by modulation of inflammation, immune response, and events leading to pulmonary fibrosis.

Our data collectively demonstrate the potent protective effects of G-003M against radiation-induced lung injury. The formulation has significantly attenuated oxidative/nitrosative stress and downregulated the expression of inflammatory/fibrogenic cytokines (Figure 9). This has occurred due to significant antioxidant and anti-inflammatory property of G-003M. Both the characteristic of our formulation has indirectly assisted in preserving immune system of animals as shown by a balanced level of inflammatory cytokines. Prolonged survival has also supported minimal pathogen advancement due to strong immune response retained in G-003M-pretreated mice. Immune modulation, anti-inflammatory, and antioxidant property of our formulation has been the principle cause in inhibiting radiation-induced lung pneumonitis and fibrosis in mice and extending their survival in disease free conditions. In our opinion, this formulation has a scope for its trial in human patients who have to undergo thoracic region radiotherapy or accidentally exposed to radiation either locally to pulmonary region or whole body.

## Ethics Statement

This study was proved by Institutional Animal Ethics Committee (INM/IAEC/16/21), INMAS, Delhi, India.

## Author Contributions

Conceived designed and performed the experiments: SV, BK, and SB. Analyzed data: SV and BK. Wrote manuscript: SV and MG. Manuscript editing: HP. Administrative support: AS.

## Conflict of Interest Statement

The authors declare that the research was conducted in the absence of any commercial or financial relationships that could be construed as a potential conflict of interest.
